# Volvulus du grêle sur mésentère commun incomplet - une redoutable complication rare chez l'adulte: à propos de 1 cas

**DOI:** 10.11604/pamj.2015.20.157.4182

**Published:** 2015-02-19

**Authors:** Mahamadoun Coulibaly, Brahim Boukatta, Ali Derkaoui, Hicham Sbai, Abdelmalek Ousadden, Nabil Kanjaa

**Affiliations:** 1 Service d'Anesthésie-Réanimation Polyvalente, CHU Hassan II de Fès, Maroc; 2 Service de Chirurgie Viscérale, CHU Hassan II de Fès, Maroc

**Keywords:** Volvulus du grêle, mésentère commun, tube digestif, Volvulus of the small intestine, common mesentery, digestive tract

## Abstract

Le mésentère commun résulte d'une anomalie de rotation du tube digestif. Il est caractérisé par la persistance d'une disposition anatomique embryonnaire secondaire à une anomalie de rotation de l'anse ombilicale primitive, constituant ainsi un méso commun à toute l'anse intestinale et une racine du mésentère extrêmement courte. Cette insuffisance de rotation est le plus souvent associée à un défaut d'accolement. Ces anomalies de rotation intestinale peuvent aboutir à des complications redoutables parfois mortelles, qui surviennent généralement au cours de la période néonatale où à l’âge pédiatrique. On estime que la prévalence de ces malformations congénitales à l’âge adulte est de l'ordre de 0,2% à 0,5% âge auquel elles demeurent très souvent asymptomatiques et donc non diagnostiquées. Le diagnostic de volvulus total du grêle peut se faire dans des circonstances très variées: en urgence devant un tableau d'occlusion intestinale aiguë, voire un état de choc pouvant conduire au décès, devant un tableau de douleurs abdominales répétées plus ou moins associées à des troubles du transit. Nous rapportons l'observation d'un patient de 18 ans admis pour volvulus total du grêle sur mésentère commun incomplet chez qui l’évolution était favorable.

## Introduction

Le mésentère commun résulte d'une anomalie de rotation du tube digestif. Il est caractérisé par la persistance d'une disposition anatomique embryonnaire secondaire à une anomalie de rotation de l'anse ombilicale primitive, constituant ainsi un méso commun à toute l'anse intestinale et une racine du mésentère extrêmement courte [1]. Cette insuffisance de rotation est le plus souvent associée à un défaut d'accolement. Ces anomalies de rotation intestinale peuvent aboutir à des complications redoutables parfois mortelles, qui surviennent généralement au cours de la période néonatale où à l’âge pédiatrique. Le fait que cette pathologie soit exceptionnelle à l’âge adulte et que sa symptomatologie soit assez variée est source de beaucoup d'erreurs et de retard diagnostique et thérapeutique au point que la majorité des cas sont diagnostiquer en post-mortem. Les causes de sa révélation tardive sont encore inconnues. La complication la plus redoutable est le volvulus total du grêle, elle survient lorsque le type de l'anomalie de rotation est le mésentère commun incomplet à 180° [2].

## Patient et observation

**Figure 1:**
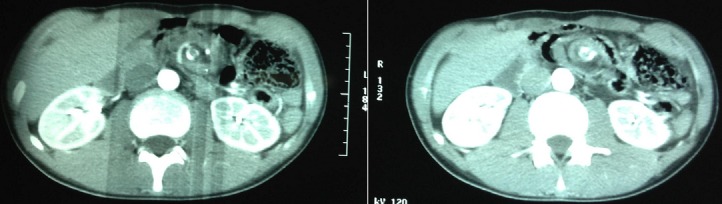
TDM montrant l'image en « tourbillon »

**Figure 2:**
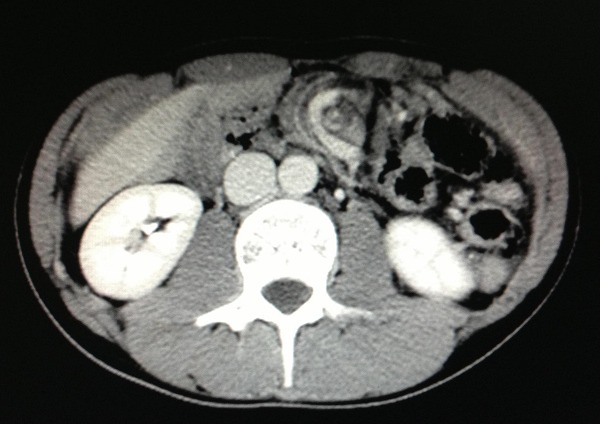
TDM montrant image « Tourbillon » et distension colique

**Figure 3:**
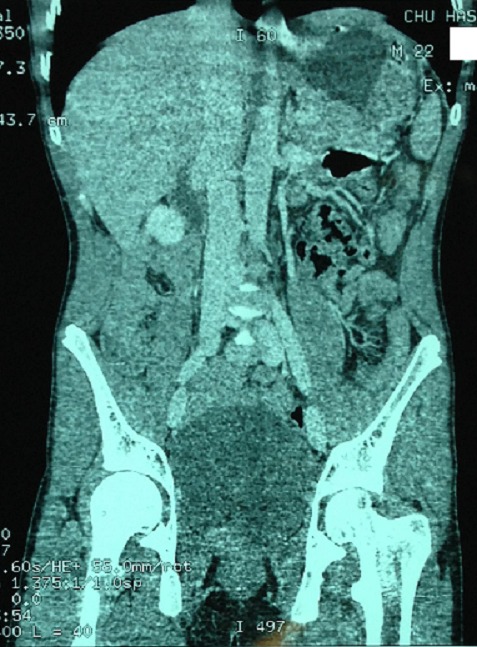
TDM, coupe coronale

**Figure 4:**
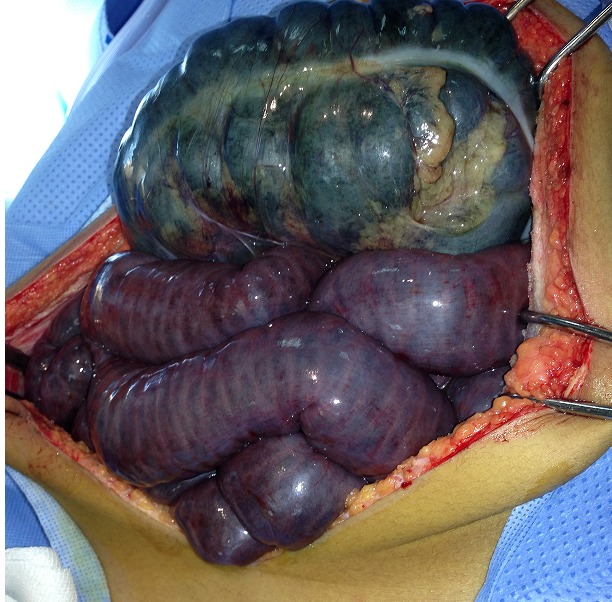
Image per-op montrant ischémie totale du grêle et du colon transverse

**Figure 5:**
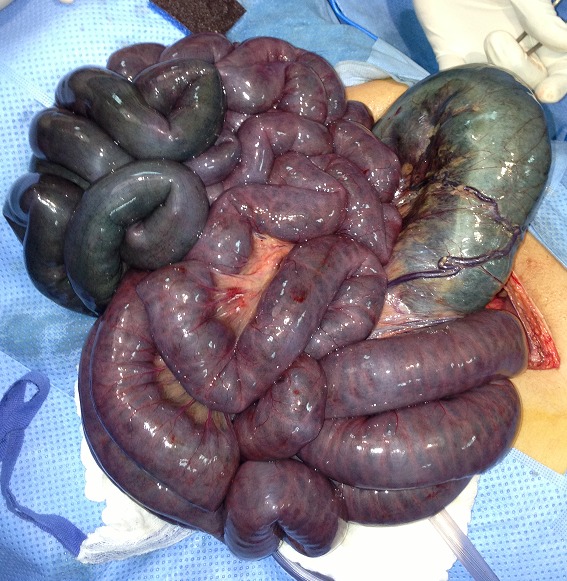
Image per-op montrant ischémie totale du grêle et du colon transverse

**Figure 6:**
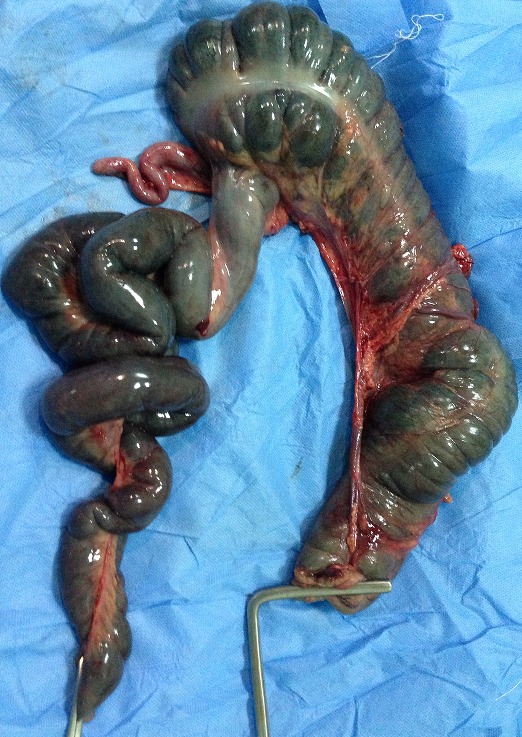
Pièce de résection opératoire

**Figure 7:**
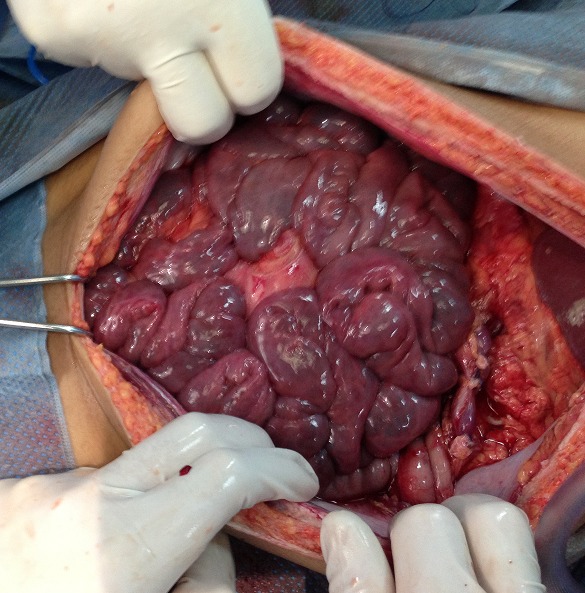
Recoloration des anses après dévolvulation

Nous rapportons l'observation d'un adolescent de 18 ans sans antécédent pathologique connu admis dans notre formation dans un tableau de syndrome occlusif, la symptomatologie évoluait depuis plus de 24 heures avant son admission et était faite de douleur abdominale d'intensité rapidement progressive avec des vomissements initialement alimentaire puis bilieux, le tout évoluant dans un contexte d'apyrexie et d'altération de l’état général. L'examen à son admission trouvait un patient en état de choc: pression artérielle à 80mmHg/30mmHg; une fréquence cardiaque à 135 battements/min; polypneique à 35 cycles/minutes; hypotherme à 36°c; somnolence avec un GCS à 12. Par ailleurs l'examen abdominal trouvait un abdomen distendu, tympanique avec contracture généralisée. Après mis en condition en déchoquage (Monitorage, oxygénothérapie au masque à haute concentration, prise d'une voie veineuse centrale fémorale droite, mis en place d'une sonde gastrique et vésicale)à noter qu'il était anurique et avait une stase gastrique importante;il a bénéficié d'un remplissage vasculaire par du sérum salé physiologique 20cc/kg en 30 minutes et devant la non réponse au remplissage vasculaire, mis sous Noradrénaline à 0.3mcg/kg/min et réalisation en parallèle d'un bilan biologique et radiologique. Le bilan Biologique trouvait une Hyperleucocytose à 24 000 éléments/mm3 à prédominance PNN avec une fonction rénale correcte, la CRP était à 135 mg/l. L’échographie abdominale était non concluante à part un écran gazeux énorme qui gênait l'examen d'où la réalisation d'une TDM abdominale C- /C+ qui trouvait une image en tourbillon intéressant la première anse jéjunal, l'artère mésentérique supérieure et la veine mésentérique superieure,avec le coecum accolé en sous hépatique et les anses grélique à droite (Figure 1, Figure 2, Figure 3) le diagnostic d'occlusion sur mésentère commun incomplet était posé et le patient fut admis d'urgence au bloc Opératoire sous couverture antibiotique à base de ceftriaxone et de métronidazole. L'exploration trouvait tout le grêle ainsi que le colon ascendant et transverse souffrant (Figure 4, Figure 5) le geste a consisté à une devolvulation (recoloration partielle du grêle), cure de l'anomalie de rotation selon la procédure de LADD et réalisation d'une colectomie subtotale droite emportant le grêle nécrosé (Figure 6) et confection de stomies (bout distal grélique à droite et bout colique à gauche). Le résultat per-opératoire était satisfaisant (recoloration partielle des anses (Figure 7). Le patient a été extubé en réanimation après stabilité hémodynamique. L’évolution était favorable (stomies normo colorées et viables), sevré de la Noradrénaline et transféré au service de chirurgie viscérale après 3 jours d'hospitalisation en réanimation.

## Discussion

On estime que la prévalence de ces malformations congénitales à l’âge adulte est de l'ordre de 0,2% à 0,5% [3, 4], âge auquel elles demeurent très souvent asymptomatiques et donc non diagnostiquées. Chez ces patients asymptomatiques, le diagnostic peut être révélé au cours de crises d'appendicite ectopique [5] ou de manière fortuite au cours d'un examen radiologique. Les complications des anomalies de rotation intestinales peuvent être aigues ou chroniques chez l'adulte, les complications évolutives aigues comprennent les occlusions duodénales par bride ainsi que le volvulus total du grêle qui reste exceptionnel chez l'adulte et dont le pronostic est redoutable. Les complications chroniques résultent des sténoses duodénales incomplètes ou de volvulus chroniques du grêle avec insuffisance artérielle mésentérique. Le diagnostic de volvulus total du grêle peut se faire dans des circonstances très variées:en urgence devant un tableau d'occlusion intestinale aiguë, voire un état de choc [6] pouvant conduire au décès, devant un tableau de douleurs abdominales répétées plus ou moins associées à des troubles du transit; plus rarement, au décours d'une chirurgie laparoscopique, comme cela a été décrit après une cholécystectomie [7–11], une appendicectomie [12, 13] ou une chirurgie de l'obésité.

L'abdomen sans préparation (ASP) peut être extrêmement variable et ne montre aucun signe spécifique, cependant il est rarement normal et généralement interpréter comme « inhabituel » ou discordant. L’échographie doppler est souvent gênée par les gaz et n'est pas toujours contributive au diagnostic, cependant sa sensibilité serait de 86,5%, sa spécificité de 74,7%, sa valeur prédictive positive de 42,1% et sa valeur prédictive négative de 96,3% [14]. Enfin, selon certains auteurs [15], l’échographie serait l'examen de référence pour éliminer une AR, lorsque celle-ci montre la présence du troisième duodénum en arrière de l'artère mésentérique supérieure. L'examen de référence pour le diagnostic du volvulus total du grêle sur anomalie de rotation intestinale chez l'adulte est la tomodensitométrie abdomino-pelvienne avec injection de produit de contraste [16–19], décrit par Fischer [20] en 1981 sous le nom de whirl-like pattern, le signe du « tourbillon » semble être pathognomonique pour la majorité des auteurs. Il correspond à la vrille du mésentère visible en position médiane, en avant de l'aorte et au niveau de l'artère mésentérique supérieure, autour de laquelle viennent « s'enrouler » la veine mésentérique supérieure et le jéjunum proximal. Le traitement du volvulus aigu du grêle sur malrotation intestinale est une urgence chirurgicale. La procédure de Ladd reste la référence [2], aussi bien chez l'adulte que chez l'enfant. Elle consiste en une laparotomie médiane suivie d'une réduction du volvulus par détorsion (dans un sens antihoraire le plus souvent), d'une section des brides responsables du raccourcissement de la racine mésentérique, d'une fixation de l'intestin en mésentère commun complet pour éviter toute récidive et enfin d'une appendicectomie de principe. L’évolution est alors généralement favorable, à condition que le diagnostic et la prise en charge thérapeutique aient été effectués rapidement.

## Conclusion

Complication redoutable et exceptionnelle à l’âge adulte. La lourde mortalité du au retard diagnostique impose la connaissance de ces anomalies de rotations ainsi que les complications qu'elles peuvent engendrées à chaque praticien. La symptomatologie clinique étant non spécifique, la réalisation d'examen radiologique ne doit accusée d'aucun retard. Le pronostic du volvulus total du grêle est celui du syndrome occlusif, de la pullulation microbienne qu'il occasionne et dépend fortement du délai de prise en charge et du terrain.
